# Benthic Microalgae Respond More Strongly to Warming and Salinity Than Zooplankton

**DOI:** 10.1002/ece3.73754

**Published:** 2026-06-02

**Authors:** Leena Virta, Jonna Engström‐Öst

**Affiliations:** ^1^ Tvärminne Zoological Station University of Helsinki Hanko Finland; ^2^ Norwegian Institute for Nature Research Fram Centre for Climate and the Environment Tromsø Norway; ^3^ Novia University of Applied Sciences Ekenäs Finland

**Keywords:** biodiversity, biomass, community, diatoms, functional traits, multiple stressors

## Abstract

The effects of elevated temperature are pronounced in high latitudes where warming is faster than the global average. It is unclear how warming and other simultaneous stressors will affect the functioning of trophic levels. To study the effects of increasing temperature and decreasing salinity on diversity, functional traits, and biomass of Baltic Sea benthic diatoms and zooplankton, we conducted an indoor mesocosm experiment. We hypothesized that higher trophic levels, that is, zooplankton, would be more vulnerable to climate change than lower trophic levels, that is, benthic diatoms, because benthic diatoms typically have a high species richness that promotes high resilience and functional redundancy. We also hypothesized that the diversity and biomass of both trophic levels would decline when subjected to warming and decreasing salinity. The study organisms were subjected to ambient temperature and +5°C warming, and five salinity treatments with targeted salinity levels between 3 and 7. Main results showed that warming affects the diversity of both taxa, as warming decreased the species and trait diversity of benthic diatoms but increased the species and trait diversity of zooplankton. The interaction of warming and decreasing salinity had significant effects on benthic diatoms. No effect of salinity on zooplankton was found. Our results emphasized the effect of multiple stressors on aquatic communities but did not support our hypothesis of stronger effects of warming and decreasing salinity on higher trophic levels. This could be due to Baltic zooplankton being well adapted to the salinities present in the area, whereas salinity ~5 is a threshold for benthic diatoms.

## Graphical abstract

The effects of multiple simultaneous stressors on different trophic levels are poorly known. Warming changed the composition of both benthic diatom and zooplankton communities, but the interaction of warming and decreasing salinity affected only benthic diatoms.

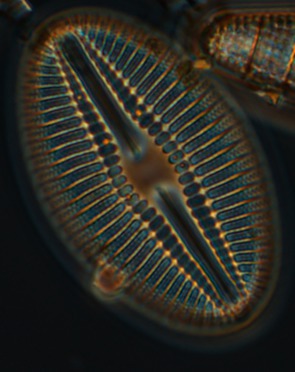

## 1 Introduction

Global climate change, induced by anthropogenic actions, is currently changing the living conditions of all organisms, and particularly the conditions of organisms living in high latitudes. This is because temperature rise will be most pronounced in high latitudes, in the Arctic even four times faster than the global average (Rantanen et al. 2022). Rising temperature will lead to severely altered functioning of high latitude marine ecosystems due to, for example, decreasing snow and ice cover, increasing frequency of extreme events, such as heatwaves and storms, and decreasing coastal salinity because of increasing precipitation (Laufkötter et al. 2020; IPCC 2023).

As the global climate is currently warming, marine heatwaves are becoming globally more common, and their intensity and duration are increasing (Forster et al. 2024; Zervoudaki et al. 2024); therefore, there is a strong need for improved understanding of both short‐ and long‐term heat stress and its consequences for coastal ecosystems (Staniek et al. 2025). Warming and heatwaves affect all organisms in the surface and sub‐surface layers of oceans, and the effects on organisms can be catastrophic, such as widespread mortality, disrupted and altered food webs, abundance shifts, and general loss of diversity (Albano et al. 2021; Penn and Deutsch 2022; Zhang et al. 2023). However, our knowledge on the responses of organisms belonging to different trophic levels is limited. It has been shown in terrestrial ecosystems that species belonging to higher trophic levels are more vulnerable to climate change than species of lower trophic levels (da Silva et al. 2023), but the effects of warming on marine organisms of different trophic levels are poorly known. Here, we address this knowledge gap by studying the effects of simultaneous stressors, that is, warming and decreasing salinity which is another predicted effect of climate change in coastal ecosystems, on the abundance, biomass, and functional traits of benthic diatoms and zooplankton.

Benthic diatoms are a major organism group in the microphytobenthic communities that inhabit all substrates in the illuminated water layer of coastal oceans. Benthic diatoms are very productive and extremely important for the functioning of marine ecosystems, as they form the basis of benthic food webs, recycle nutrients, stabilize sediments hence forming habitats for other organisms, and participate in the long‐term sequestration of carbon. The extremely high species richness of benthic diatom communities increases their resilience to environmental change, but it seems that their cell size can decrease during warming, decreasing salinity and associated changes in nutrient availability (Svensson et al. 2014). As benthic diatoms are the main food source for benthic grazers (Guo et al. 2016), decreasing diatom cell size is crucial for the benthic food webs. However, the effects of warming on benthic diatom communities and their functioning are poorly known (but see Engel et al. 2020). Zooplankton are major consumers and prey for higher trophic levels in the marine ecosystem. Apart from changing phenology and range shifts, the most well‐known effect of global warming on zooplankton is the decrease in body size (Mäkinen et al. 2017). Daufresne et al. (2009) demonstrated strong effects on bacteria, plankton and fish as the proportion of small‐sized zooplankton species and young age classes increased and the size‐at‐age decreased. In addition, survival, growth and reproduction, as well as physiological processes, are affected by elevated temperature (reviewed by Ratnarajah et al. 2023).

Biodiversity is most often described by species abundances, but linking diversity to ecosystem functioning can be much more effective when using functional traits (Hooper et al. 2005). Traits can be described as main characterizing agents of phenotype, functions and performance (Arnold 1983). While traits describe species performance, responses and interactions, quite a few approaches link them directly to ecosystem processes and changes (Passy 2007; Hébert et al. 2017). The usefulness of the trait‐based approach has also been shown in biodiversity research regarding benthic diatoms and zooplankton. For benthic diatoms, key traits include, for example, cell size, life‐form, and colony‐forming abilities, and Virta et al. (2019) showed that functional traits are more useful than species abundances for revealing the relationship between diatom diversity and ecosystem productivity. For zooplankton, key traits are, for example, reproductive mode, body size, and feeding mode, and traits can be highly useful for characterizing changes in the zooplankton community during environmental change (Litchman et al. 2013).

The Baltic Sea is a relatively small, semi‐enclosed sea in northern Europe. Due to its small size, shallow depth, and location in high latitudes, it is currently experiencing strong climate‐change related changes. The Baltic Sea is among the seas that are warming most rapidly on Earth (Reusch et al. 2018), and the salinity of the Baltic Sea is decreasing due to increasing run‐off from rivers and less frequent saline water inflows from the North Sea. Because of the rapidness of these changes, the Baltic Sea can be used as a “time‐machine” for other oceans, in terms of understanding and explaining the fast climate‐change related processes in the ecosystem.

Here, our aim was to investigate the responses of different trophic levels to climate change using the communities of benthic diatoms and zooplankton of the Baltic Sea as model groups. We hypothesized that (H1) lower trophic levels, that is, benthic diatoms, are less vulnerable to climate change than higher trophic levels, that is, zooplankton. This means that the species and trait community composition and diversity of lower trophic levels show less variability in the face of warming and decreasing salinity. This could be due to higher species richness and consequent higher trait redundancy in the lower trophic level resulting in the maintenance of high trait diversity despite changing species composition, and a larger seed bank of the lower trophic level leading to an effective replacement of disappearing species (Zobel 1997), and (H2) the diversity and biomass of both trophic levels decline when confronted with elevated temperature and increase when confronted with decreasing salinity (Remane 1934; Cartaxana et al. 2015), but the changes are smaller in lower trophic levels, that is, benthic diatoms (Kwiatkowski et al. 2018).

## 2 Materials and Methods

### 2.1 Experimental Set‐Up

**FIGURE 1:**
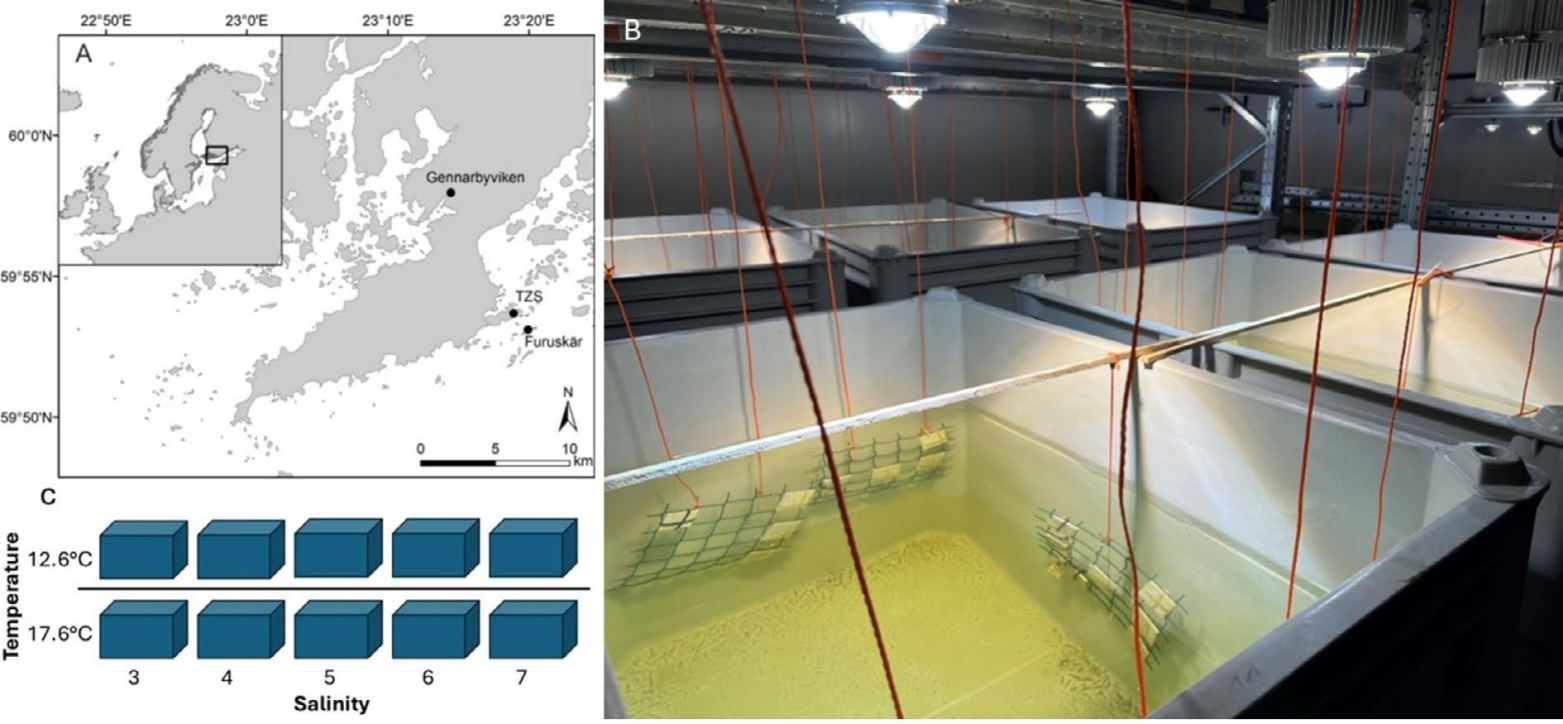
(A) Map of the island (Furuskär) where the artificial substrates were incubated in the sea for diatom colonization, Gennarbyviken that was our freshwater source, and Tvärminne Zoological Station (TZS), where the mesocosm experiment was conducted, (B) mesocosms with artificial substrates (tiles) attached to nets, and (C) the mesocosm experiment design with two temperature levels and five salinity levels.

We conducted the experiment during May–June 2023 using indoor mesocosms at the Tvärminne Zoological Station, Hanko, Finland. Prior to the start of the experiment, we attached artificial substrates (ceramic tiles ~5 cm × ~5 cm) to nets and placed them in the sea at Furuskär island in Hanko archipelago, ~0.5 m below the surface to ensure photic conditions for six weeks for colonization of benthic diatom communities (Hillebrand and Sommer 2000; Figure 1). The experiment consisted of 10 plastic enclosures (volume 600 L) and combined two temperature levels (~12.6°C which was approximately the temperature in the sea in the beginning of the experiment, and ~17.6°C; Laufkötter et al. 2020) and five salinity levels (target salinities 3, 4, 5, 6, and 7; salinity in the sea at the sites where our artificial tiles were placed for colonization and where 2/3 of the mesocosm water originated from was approximately 6).

On May 24, we filled each mesocosm with ~200 L of water collected in Gennarbyviken reservoir (salinity ~0), which is the closest freshwater source, and with ~400 L of water collected from the bay in front of Tvärminne Zoological Station (salinity ~6). On May 24 and 25, we added sea salt (Aquarium System Instant Ocean) to each mesocosm to produce five different salinity treatments. Formula m_salt_ = (S_target_—S_initial_) × V, where m_salt_ = mass of salt to add (g), S_target_ = desired salinity (g/kg or PSU), S_initial_ = starting salinity, and V = volume of water (L), was used for the salt additions. Salt additions were done by extracting ~10 L of water from each mesocosm, mixing salt in the extracted water until it was completely dissolved, and by adding the salt water into the mesocosm using Secchi discs to ensure proper mixing. Mesocosms were placed in two different rooms of 12.6°C and 17.6°C as room air temperatures, respectively, to achieve the temperature treatments. Light was supplied by a separate lamp (LEDspot 200W flex 90°, made by Aqua Medic) above each mesocosm. The lights were controlled by a computer simulating daily irradiance curves, with sunrise at 4.32 a.m., sunset at 22.18 p.m., and dimming following the daily light curve.

On May 25, we transported the artificial substrates (tiles) from the sea to the mesocosms in boxes filled with seawater. We attached nets with tiles vertically into the mesocosms to avoid the accumulation of dead material on the tiles. During the experiment, we measured temperature and salinity in each mesocosm with a portable calibrated digital water meter (YSI Prosolo multiparameter meter) twice a week. At the beginning and end of the experiment, we collected water from each mesocosm for nutrient analyses (${\mathrm{NO}}_{2}^{-}$ + ${\mathrm{NO}}_{3}^{-}$ and ${\mathrm{PO}}_{4}^{3-}$) that were run with a Thermo Scientific Aquakem 250 nutrient analyzer (Thermo Fisher Scientific Oy, Vantaa, Finland). The duration of the entire mesocosm experiment was 4 weeks.

### 2.2 Sampling and Characterization of Species, Traits, and Biomass of Benthic Diatoms

We collected diatom samples from tiles before transporting the tiles to mesocosms, and at 1, 2, 3, and 4 weeks after starting the treatments. Each time, diatom samples were collected from six tiles per mesocosm (except before the transport of tiles to mesocosms, when six tiles altogether were sampled). Three of these six tiles were used for the identification of community composition of diatoms for which samples were collected by rubbing one side of the tile with a sponge (~2 cm × 2 cm × 2 cm) until the entire biofilm was collected. A new sponge was used for each tile, and the sample collected from each tile was stored in a separate container. Samples were preserved in ethanol and stored in cold (+6°C) and dark conditions. These diatom samples were boiled in hydrogen peroxide (30% H_2_O_2_) to remove organic material, cleaned diatoms mounted on slides using Naphrax (Brunel Microscopes, Chippenham, UK), and 500 valves per sample identified to the lowest possible level (typically species level) with a phase contrast light microscope using 1000× magnification. Species identification followed Krammer and Lange‐Bertalot (1986, 1988, 1991a, 1991b), Snoeijs (1993), Snoeijs and Kasperovicienè (1996), Snoeijs and Potapova (1995), and Snoeijs and Vilbaste (1994). After the identification, we transformed species counts to relative abundances and verified taxonomic names using AlgaeBase (Guiry and Guiry 2025).

The functional community composition was accounted for by using the abundances of traits. Each species was classified with all the following six traits: (1) size (large: > 1000 μm^3^/small: < 1000 μm^3^), (2) mobility (mobile [with a raphe]/non‐mobile [without a raphe]), (3) type of attachment (adnate/pedunculate [stalk‐attached/pad‐attached]/non‐attached), (4) ability to form colonies (does form colonies/does not form colonies), (5) guild (low‐profile/high‐profile/motile/planktonic; Rimet and Bouchez 2012), and (6) nitrogen‐fixing abilities (nitrogen‐fixer/non‐nitrogen‐fixer; Passy 2007). For trait identification, we used the above‐mentioned species literature as well as Diatoms of North America (Spaulding et al. 2021) and Snoeijs et al. (2002). The trait composition of a community was calculated as the trait combination of all species present in the community.

The remaining three tiles per mesocosm were used for measuring the biomass of the biofilm on tiles. All material from the upper side of each tile was scraped with a sharp blade. Half of this material was stored on a pre‐scaled petri dish, dried (60°C, 12 h) and scaled again for biomass. The other half was stored for later use. It should be noted that biofilm on the tiles consists of several organism groups, both photosynthesizing, such as diatoms and cyanobacteria, and heterotrophic, such as heterotrophic bacteria and archaea. Therefore, the biomasses measured from the tiles are not solely provided by diatoms. However, diatoms are the dominating group of microphytobenthos in the Baltic Sea when water temperature is below ~20°C (Watermann et al. 1999), and thus, we think that the biomasses measured from the tiles are good proxies for the biomass of benthic diatoms.

### 2.3 Sampling and Characterization of Species, Traits, and Biomass of Zooplankton

Zooplankton samples were collected in the beginning of the experiment, during week 3, and in the end (30 samples in total). Each container was mixed using a Secchi disk before collecting 5 L of mesocosm water, concentrating it using a 48 μm sieve, and preserving the concentrate with acid Lugol's solution in plastic 50 mL Falcon tubes. Samples were stored in the fridge until analysis. Micro‐ and mesozooplankton (except ciliates) were identified from the whole sample using a Bogorov counting chamber under a Leica microscope at 30× magnification. Taxa were identified to species or genus level (Rajasilta and Vuorinen 2008). Counted taxa belong to Cladocera, Copepoda, and Rotifera.

For zooplankton, life‐history traits were noted by using the abundances of traits, similarly as for diatom community. Each species or genus was classified to four traits: (1) body size (microzooplankton < 200 μm/mesozooplankton > 200 μm), (2) feeding mode (herbivorous/omnivorous/carnivorous), (3) reproductive mode (external egg sac/internal egg sac/broadcast spawner), and (4) resting stages (resting stages present/absent) (Litchman et al. 2013; Hébert et al. 2017). The trait composition of a community was calculated as the trait combination of all species present in the community. Copepod nauplii (juveniles) were not classified for the reproductive mode and resting stage mode. All zooplankton biomasses were calculated according to Hernroth (1985).

### 2.4 Statistical Analyses

We illustrated the species and trait composition patterns of benthic diatom and zooplankton communities with pie charts and Nonmetric Multidimensional Scaling (NMDS). In NMDS, we used Bray–Curtis distance and two (diatom traits, zooplankton species and traits) or three (diatom species) dimensions. Community compositions of diatoms in different treatments were calculated as averages of compositions in all three samples from that treatment. We used R packages *vegan* (Oksanen et al. 2025), *ggplot2* (Wickham 2016), and *ggpubr* (Kassambara 2023).

To illustrate the abundance of benthic diatom and zooplankton species in different treatments, we constructed heatmaps using R package *pheatmap* (Kolde 2019). We used Euclidean distance as a similarity measure. Abundances in different treatments were calculated as averages of abundances in all samples from that treatment, and the abundances were log10 + 1‐transformed. For diatoms, only the 10 most abundant species were used.

We analyzed differences in the species and trait composition of benthic diatoms and zooplankton between different salinities and temperatures by calculating the analysis of similarities (ANOSIM) with Bray–Curtis distance (Bray and Curtis 1957). Before analysis, we transformed community data into dissimilarity indices using function *vegdist* in R package *vegan* (Oksanen et al. 2025). In ANOSIM, values close to 1 denote greater dissimilarity between groups than within groups, and values close to −1 denote greater dissimilarities within groups than between groups.

We analyzed the effect of salinity, temperature, salinity × temperature interaction, and time on species and trait composition of benthic diatom and zooplankton communities during the entire experiment and 1, 2, 3, and 4 weeks after the beginning of the experiment using permutational multivariate analysis of variance (PERMANOVA) in R package *vegan* (Oksanen et al. 2025). Analyses for species composition were performed with 999 permutations and the Bray–Curtis dissimilarity index, and analyses for trait composition with 999 permutations and the Gower dissimilarity index (Bray and Curtis 1957; Gower 1971). Community compositions of diatoms in different treatments were calculated as averages of compositions in all three samples from that treatment. We assessed homogeneity of multivariate dispersion using PERMDISP (betadisper function) based on experimental treatment groups.

As a measure of species and trait diversity of benthic diatom and zooplankton communities, we used Shannon's diversity index (Shannon 1948). Diversity and biomass of diatoms in different treatments were calculated as average values of all three samples from that treatment. We analyzed the effect of salinity, temperature, and their interaction on the diversity and biomass of communities using linear models. Salinity was treated as a continuous variable and temperature as a categorical variable. To investigate direct and indirect relationships among temperature and salinity, species and trait diversity of benthic diatom and zooplankton communities, and biomass, we used piecewise structural equation models (SEMs) with R package *piecewiseSEM* (Lefcheck 2016). We constructed initial full models of hypothesized paths between all variables and considered temperature and salinity as exogenous variables, that is, variables that exist only as predictors in the network. Initial models for benthic diatoms included a path from diatom diversity to biomass, because diversity and biomass were calculated with separate measurements and therefore were not interdependent. For zooplankton, this path was excluded because diversity as well as biomass were calculated based on species abundances and therefore were dependent on each other. From the initial models, we eliminated nonsignificant paths stepwise. We considered models with nonsignificant *p* values (> 0.05) as candidate models and chose the models with lowest Akaike's information criterion (AIC; Akaike 1974) as the best‐fit models. All statistical analyses were conducted using R version 4.4.3 (R Core Team 2025).

## 3 Results

**FIGURE A1:**
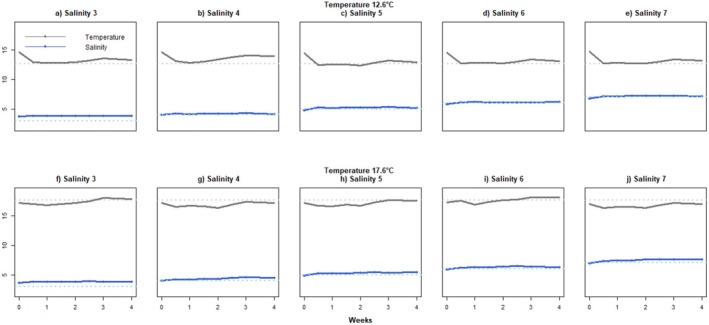
Variation of temperature (°C) and salinity in the ten mesocosms during the 4‐week‐long experiment. Panels a–e represent mesocosms in the room with target temperature 12.6°C (ambient temperature), and panels f–j represent mesocosms in the room with target temperature 17.6°C (heatwave). Gray lines denote temperature and blue lines denote salinity. Continuous lines denote the realized temperature and salinity, and dashed lines denote the target temperature and salinity.

Minor deviations from the intended salinity treatments occurred in some mesocosms, mainly in the mesocosms with target salinity 3, where the realized salinity in the beginning of the experiment was 3.8. Salinity remained stable in all mesocosms during the experiment (Figure A1, Table A1). Temperature showed some variation in all mesocosms, as temperature in the mesocosms with target temperature 12.6°C varied between 12.5°C and 13.9°C (mean 12.98°C) and temperature in the mesocosms with target temperature 17.6°C varied between 16.5°C and 18.1°C (mean 17.07°C). Temperature variation among mesocosms was due to slight air temperature variation within rooms, and the temporal temperature variation was due to changes in outside temperature that also affected the temperature in the mesocosm rooms.

Nutrient concentrations were consistent in all mesocosms. In the beginning, the concentration of ${\mathrm{NO}}_{3}^{-}$ + ${\mathrm{NO}}_{2}^{-}$ was between 28.3 and 39.9 μg/L and the concentration of ${\mathrm{PO}}_{4}^{3-}$ < 3 μg/L in all mesocosms. In the end, ${\mathrm{NO}}_{3}^{-}$ + ${\mathrm{NO}}_{2}^{-}$ and ${\mathrm{PO}}_{4}^{3-}$ concentrations were < 3 μg/L in all mesocosms (Table A2).

**FIGURE 2:**
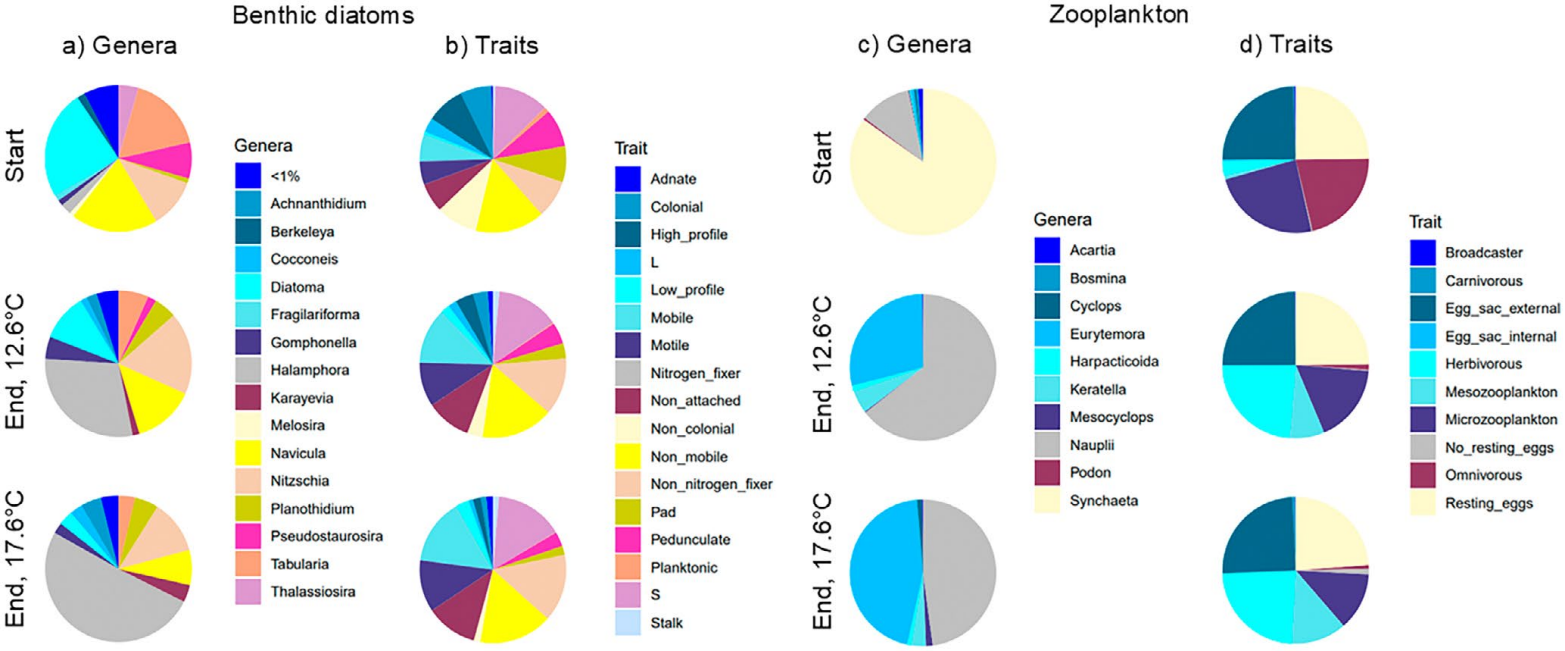
The relative abundances of (a) benthic diatom genera, (b) benthic diatom traits, (c) zooplankton genera, and (d) zooplankton traits during the start of the experiment and during the end of the experiment in ambient temperature treatment (12.6°C) and heatwave treatment (17.6°C). In diatom traits, S denotes small species (biovolume < 1000 μm^3^), and L denotes large species (biovolume > 1000 μm^3^).

**FIGURE 3:**
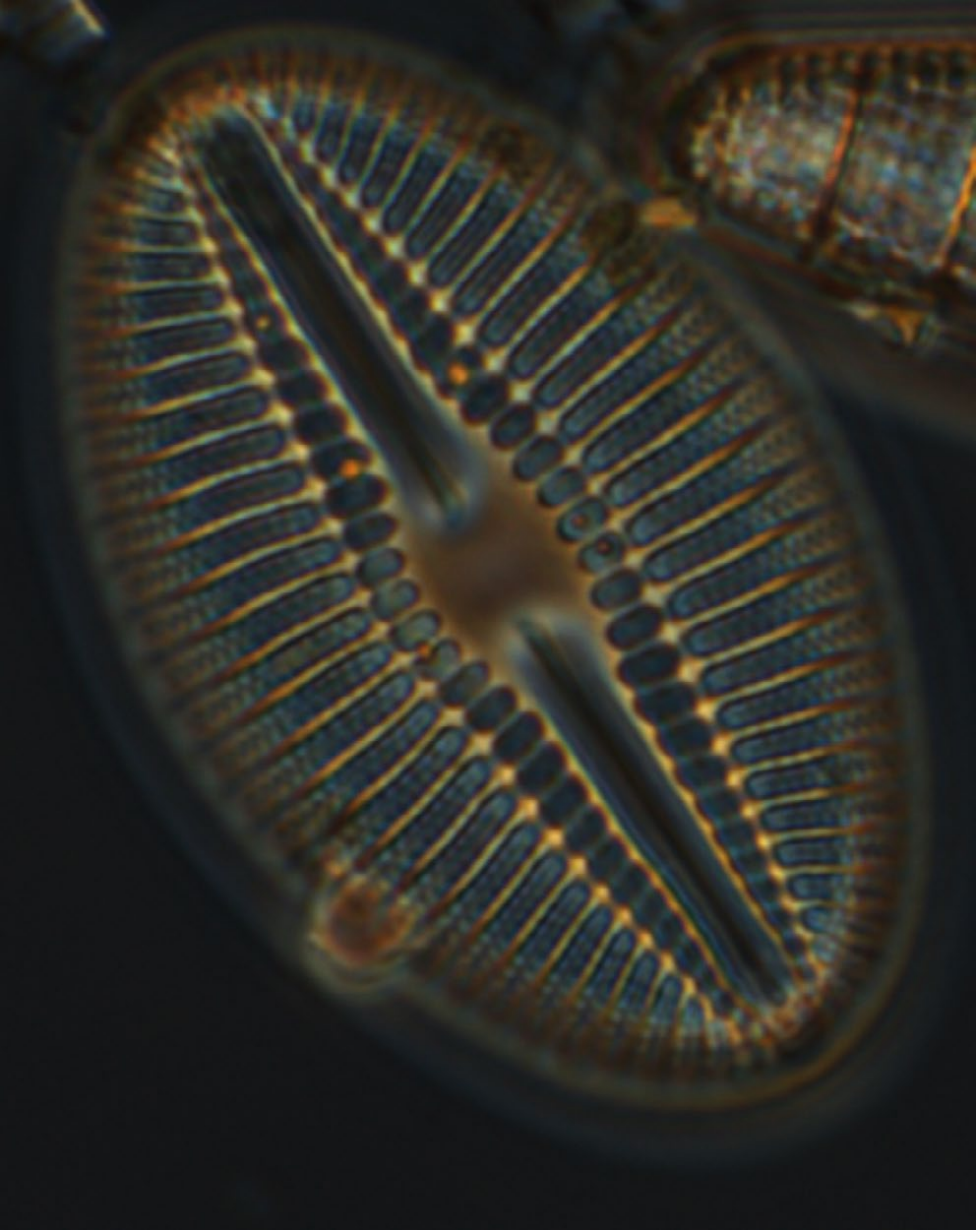
Benthic diatom *Diploneis smithii* (Brébisson) Cleve.

In total, 193 benthic diatom species and 12 zooplankton genera were identified during the experiment. Diatom species richness varied between 26 and 55 per sample, and zooplankton genus richness between 2 and 9 per sample. In the start of the experiment, the most abundant genera in the diatom communities were *Diatoma* sp., *Navicula* sp., and *Tabularia* sp. (Figure 2a). In the end of the experiment, the ambient temperature treatment was dominated by *Halamphora* sp., *Navicula* sp., and *Nitzschia* sp., whereas in the heatwave treatment, only *Halamphora* sp. was very dominant. For diatoms, all other traits were present in all samples, except the nitrogen‐fixing trait, which was only present in 12 samples. During the start of the experiment, no dominant traits could be detected in the diatom communities. In the end of the experiment, the abundances of mobile, motile, and non‐attached traits had increased (see Figure 3 for an example of a motile diatom species), and the abundances of colonial, high‐profile, pad‐attached, and pedunculate traits had decreased (Figure 2b). In the zooplankton communities, *Synchaeta* sp. was the dominant genus in the beginning of the experiment (Figure 2c). In the end of the experiment, the zooplankton communities were dominated by genus *Eurytemora* and copepod nauplii (juveniles). Traitwise, herbivorous zooplankton increased and omnivorous decreased towards the end of the experiment (Figure 2d). Groups that were affected were mesozooplankton (increasing trend) and microzooplankton (decreasing trend).

### 3.1 The Vulnerability of Organism Groups Belonging to Different Trophic Levels to the Effect of Warming and Decreasing Salinity (H1)

**FIGURE 4:**
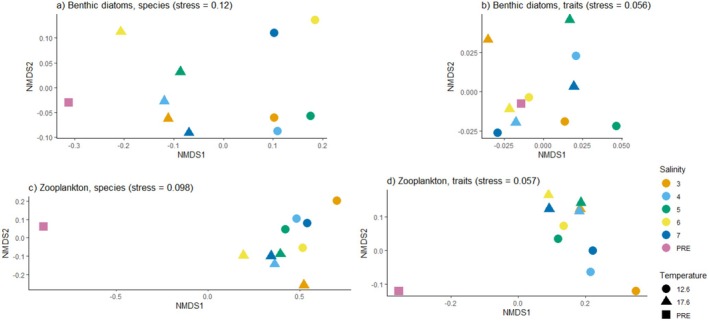
Non‐metric multidimensional scaling (NMDS) plots illustrating the species (a, c) and trait (b, d) composition of communities of benthic diatom and zooplankton communities. Each dot represents the centroid of all samples from that treatment. In the figure legend, PRE denotes samples that were collected before the start of the experiment.

NMDS revealed that different temperatures formed distinct groups of the species composition of diatom and zooplankton communities and the trait composition of zooplankton communities, along the *x*‐axis for benthic diatoms and along the *y*‐axis for zooplankton (Figure 4). Different salinities formed a clear pattern for the species composition of benthic diatom communities, where communities in salinities 3, 4, and 5 were separated from communities in salinities 6 and 7.

**FIGURE 5:**
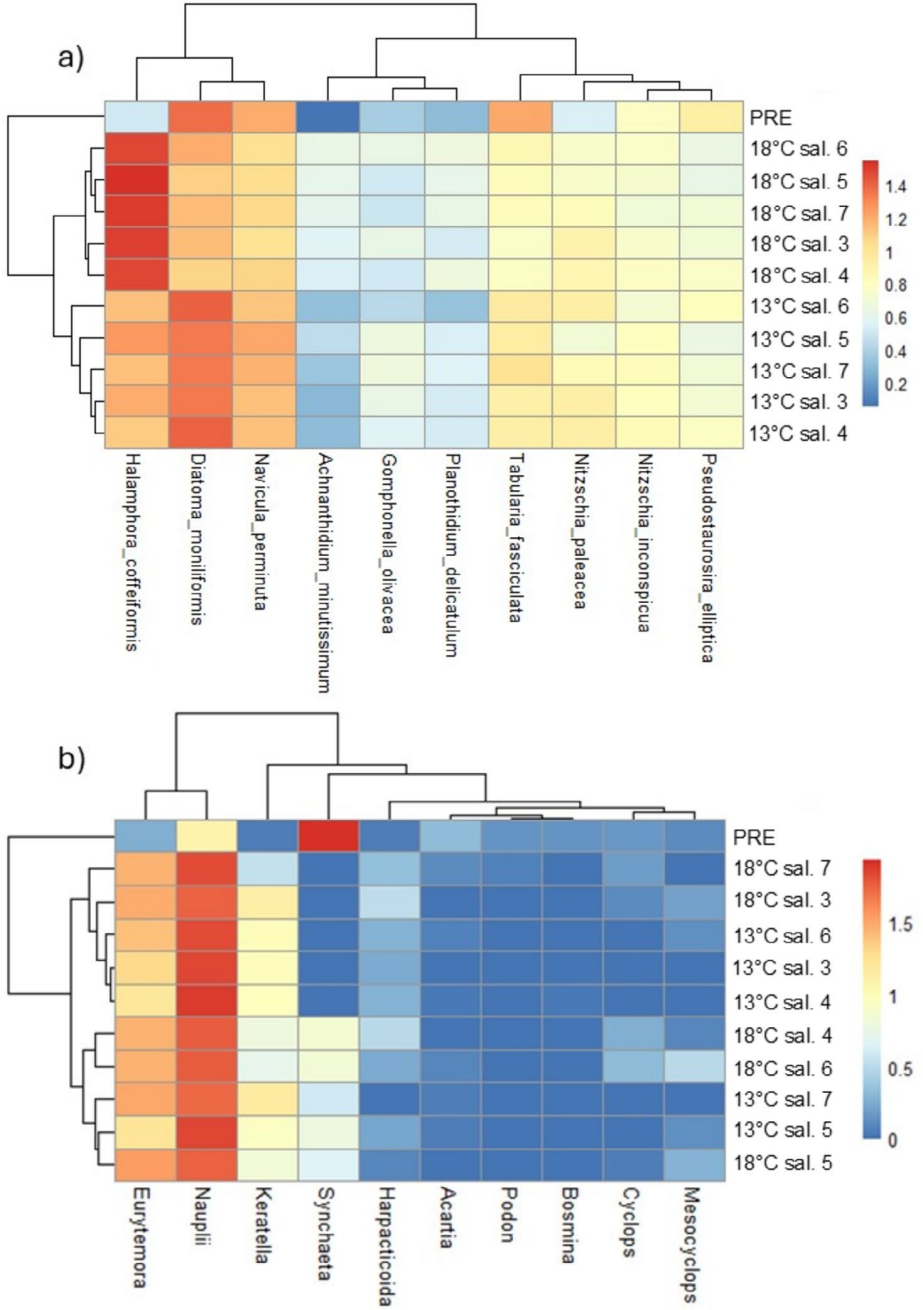
Heatmaps showing the abundance of (a) benthic diatom and (b) zooplankton species in different treatments. Relative abundances have been log 10 + 1‐transformed, and columns and rows have been rearranged based on measures of similarity. For diatoms, only 10 most abundant species are shown.

A heatmap showing the abundances of the most abundant species revealed differences between temperatures (Figure 5). For diatoms, *Halamphora coffeiformis* (C.Agardh) Mereschkowsky was the most abundant species in temperature +17.6°C, and *Diatoma moniliformis* (Kützing) D. M. Williams was the most abundant species in temperature +12.6°C and in the samples collected before the start of the treatments. The abundance of *Achnanthidium minutissimum* (Kützing) Czarnecki was very low in temperature +12.6°C and in the samples collected before the start of the treatments, but higher in temperature +17.6°C. For zooplankton, the most obvious change in the mesocosms was that a rotifer‐dominated community in all mesocosms (+12.6°C and +17.6°C) turned into a copepod‐dominated community, mostly in the higher temperature (+17.6°C), consisting of nauplii, copepodites and adults.

ANOSIM showed significant differences in the species and trait composition of benthic diatom and zooplankton communities between temperature treatments, as well as between temperature treatments and samples that had been collected before the start of the experiment (Table 1). The species composition of benthic diatom communities was also significantly different between samples collected before the start of the treatments and most of the salinity treatments, and between salinity treatments 3 and 7. No significant differences were detected in the species and trait composition of zooplankton between salinity treatments.

**TABLE 1:** Results of the analyses of similarities (ANOSIM) to analyze differences in species and trait composition of benthic diatom and zooplankton communities between (a) different temperature treatments and (b) different salinity treatments.

	Benthic diatoms				Zooplankton			
	Species composition		Trait composition		Species composition		Trait composition	
	*R*	*p*	*R*	*p*	*R*	*p*	*R*	*p*
(a) Temperature treatments								
Pre‐12.6°C	**0.456**	0.006**	0.025	0.389	**1.000**	0.002**	**1.000**	0.002**
Pre‐17.6°C	**0.605**	0.001***	**0.379**	0.001***	**1.000**	0.003**	**0.997**	0.001***
12.6°C–17.6°C	**0.285**	0.001***	**0.169**	0.001***	**0.166**	0.036*	0.129	0.060
(b) Salinity treatments								
Pre‐3	**0.265**	0.023*	0.176	0.078	1.000	0.067	1.000	0.067
Pre‐4	**0.205**	0.046*	0.050	0.26	1.000	0.067	1.000	0.067
Pre‐5	0.186	0.065	0.059	0.228	1.000	0.067	1.000	0.067
Pre‐6	**0.276**	0.039*	0.027	0.363	1.000	0.067	0.929	0.067
Pre‐7	**0.249**	0.033*	0.043	0.278	1.000	0.067	1.000	0.067
3–4	0.003	0.327	−0.015	0.595	−0.104	0.695	−0.218	1.000
3–5	0.043	0.09	−0.004	0.408	−0.052	0.604	−0.125	0.640
3–6	0.046	0.084	0.003	0.33	−0.146	0.802	−0.156	0.749
3–7	**0.068**	0.045*	−0.004	0.388	0.088	0.338	−0.104	0.630
4–5	0.015	0.201	−0.021	0.719	−0.219	0.889	−0.208	0.919
4–6	0.032	0.126	−0.003	0.403	−0.219	0.921	−0.229	1.000
4–7	0.032	0.126	−0.020	0.697	−0.021	0.512	−0.052	0.636
5–6	0.031	0.118	−0.002	0.36	−0.240	0.973	−0.135	0.710
5–7	0.016	0.201	−0.018	0.654	−0.104	0.704	−0.146	0.816
6–7	0.001	0.352	−0.023	0.783	−0.125	0.815	−0.104	0.764

*Note:* Pre denotes samples that were collected prior to starting the treatments. Significant differences are bolded. ***p ≤ 0.001; **p ≤ 0.01; *p ≤ 0.05.

PERMANOVA revealed that temperature was the most important environmental variable affecting species and trait composition of benthic diatom and zooplankton communities with a significant effect during almost all sampling weeks (Table 2). Temperature‐salinity interaction was significant for diatoms during almost all sampling weeks but always nonsignificant for zooplankton. The individual effect of salinity was nonsignificant for both organism groups. In the analyses of the entire experiment duration, the effect of time was strongest for both organism groups. No significant differences in multivariate dispersion were detected among salinity treatments (PERMDISP *F* = 0.77, *p* = 0.578), but dispersion differed significantly among temperature treatments (*F* = 7.17, *p* = 0.002). This indicates that the effect of temperature detected by PERMANOVA may reflect differences in both community composition and dispersion among treatments.

**TABLE 2:** Results of the PERMANOVA analyses to analyze the effect of temperature, salinity, temperature × salinity interaction, and time on the species and trait composition of benthic diatom and zooplankton communities during (a) the entire 4‐week‐experiment, (b) after a week (only diatoms because zooplankton were not sampled), (c) after 2 weeks (only diatoms because zooplankton were not sampled), (d) after 3 weeks, and (e) after 4 weeks since the beginning of the experiment.

Benthic diatoms								
	Species				Traits			
	Sum of squares	*R* ^2^	*F*	*p*	Sum of squares	*R* ^2^	*F*	*p*
(a) Entire experiment								
Time	1.586	0.531	44.166	0.001***	0.408	0.688	86.176	0.001***
Temperature	0.801	0.268	14.304	0.001***	0.180	0.304	17.024	0.001***
Salinity	0.026	0.009	0.338	0.701	0.000	0.001	0.021	0.935
Temperature × Salinity	0.841	0.281	4.831	0.002**	0.181	0.306	5.426	0.005**
(b) After 1 week								
Temperature	0.025	0.209	2.117	0.042*	0.002	0.273	3.003	0.113
Salinity	0.013	0.110	0.990	0.440	0.000	0.031	0.255	0.731
Temperature × Salinity	0.054	0.454	1.665	0.047*	0.001	0.375	1.201	0.397
(c) After 2 weeks								
Temperature	0.134	0.615	12.766	0.006**	0.023	0.835	40.371	0.012*
Salinity	0.009	0.043	0.357	0.833	0.000	0.007	0.057	0.888
Temperature × Salinity	0.150	0.690	4.447	0.013*	0.024	0.857	11.956	0.013*
(d) After 3 weeks								
Temperature	0.463	0.806	33.209	0.005**	0.091	0.878	57.314	0.009**
Salinity	0.017	0.029	0.241	0.738	0.000	0.001	0.005	0.979
Temperature × Salinity	0.491	0.853	11.647	0.007**	0.092	0.889	15.993	0.008**
(e) After 4 weeks								
Temperature	0.195	0.580	11.035	0.005**	0.022	0.664	15.786	0.006**
Salinity	0.015	0.043	0.361	0.760	0.000	0.014	0.112	0.848
Temperature × Salinity	0.218	0.648	3.683	0.022*	0.024	0.717	5.064	0.029*

Zooplankton								
	Species				Traits			
	Sum of squares	*R* ^2^	*F*	*p*	Sum of squares	*R* ^2^	*F*	*p*
(a) Entire experiment								
Time	4.487	0.846	153.24	0.001***	0.406	0.769	93.115	0.001***
Temperature	1.526	0.288	11.306	0.002**	0.214	0.404	18.99	0.002**
Salinity	0.312	0.059	1.751	0.179	0.021	0.040	1.154	0.298
Temperature × Salinity	0.160	0.030	0.870	0.374	0.033	0.063	1.892	0.184
(b) After 3 weeks								
Temperature	0.012	0.052	0.435	0.718	−0.000	−0.002	−0.012	0.916
Salinity	0.027	0.115	1.045	0.406	0.002	0.114	1.030	0.396
Temperature × Salinity	0.017	0.072	0.624	0.603	0.001	0.069	0.593	0.579
(c) After 4 weeks								
Temperature	0.099	0.709	19.472	0.004**	0.011	0.629	13.536	0.004**
Salinity	−0.000	−0.001	−0.004	0.969	0.000	0.013	0.105	0.928
Temperature × Salinity	0.029	0.210	2.121	0.161	0.004	0.225	2.328	0.149

*Note:* Temperature × Salinity denotes the interaction of these variables. ***p ≤ 0.001; **p ≤ 0.01; *p ≤ 0.05.

### 3.2 The Effect of Warming and Decreasing Salinity on the Diversity and Biomass of Communities (H2)

**FIGURE 6:**
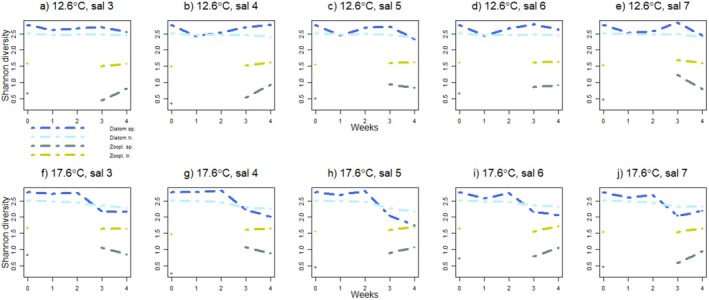
The development of species and trait diversity (Shannon's diversity index) of benthic diatom and zooplankton communities in different temperature and salinity treatments along the four‐week‐long experiment. Values for diatoms are average values of the three replicates taken from each treatment. Zooplankton samples were not collected during the first and second week of the experiment.

The species diversity of benthic diatom communities was more variable than the trait diversity along the course of the experiment (Figure 6). In most mesocosms with the temperature treatment of +12.6°C, species diversity declined during the first week, increased during the second and third week, and declined again during the fourth week, whereas trait diversity remained stable throughout the experiment and declined only slightly during the fourth week. In all mesocosms with the temperature treatment of +17.6°C, species and trait diversity of diatoms remained stable during the first and second week of the experiment but declined after that, species diversity more steeply than trait diversity.

Linear models showed no significant effects of salinity and temperature or their interaction on the diversity or biomass of communities.

**FIGURE 7:**
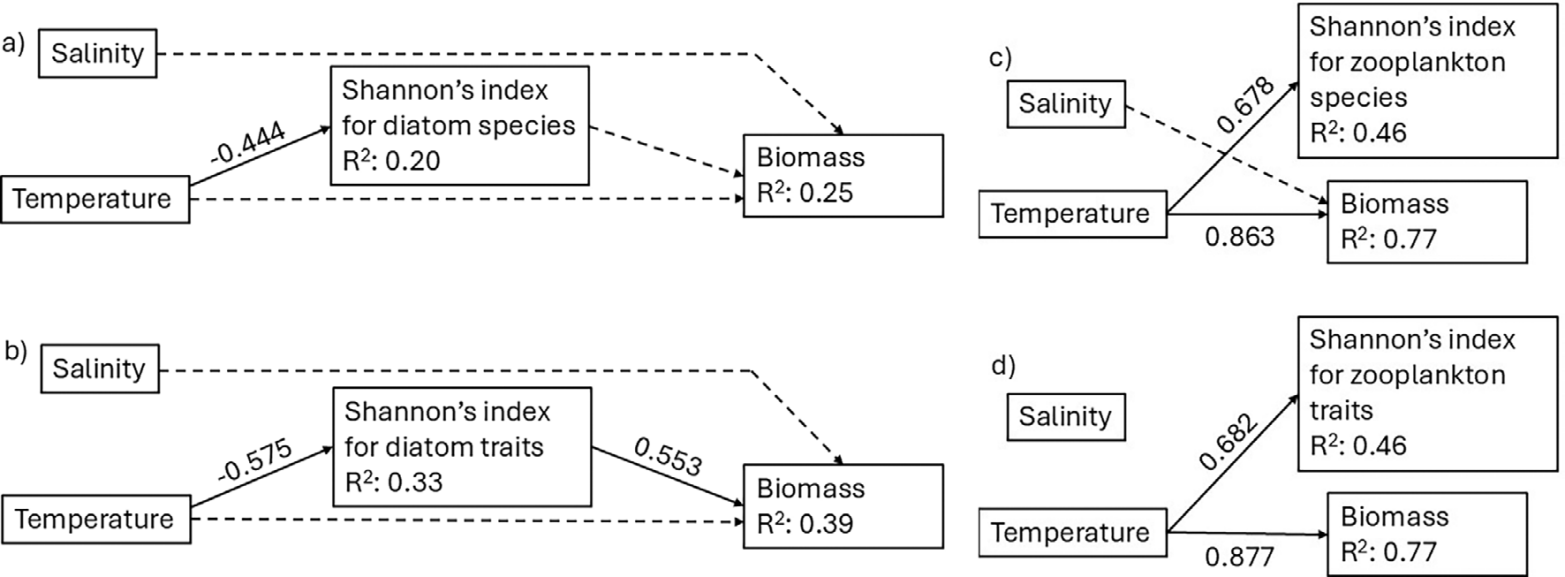
Best‐fit structural equation models (SEM) showing the relationships between salinity and temperature, Shannon's diversity index of benthic diatom species (a), benthic diatom traits (b), zooplankton species (c), or zooplankton traits (d), and biomass. The solid lines denote significant (*p* < 0.05) relationships and dashed lines denote non‐significant results (*p* > 0.05). Numbers next to lines are standardized coefficients.

The final SEM with diatom species diversity explained 25% of variation in ecosystem biomass and indicated that diatom species diversity was negatively controlled by temperature (Figure 7). The final SEM with diatom trait diversity explained 39% of variation in ecosystem biomass and indicated that diatom trait diversity was negatively controlled by temperature and biomass was positively controlled by diatom trait diversity. Other paths in the models were insignificant. The final SEMs with zooplankton diversity explained 46% of diversity and 77% of biomass and indicated that zooplankton diversity and biomass were positively controlled by temperature.

## 4 Discussion

Climate change is severely altering the living conditions of aquatic ecosystems, but differences in the responses of organisms belonging to different trophic levels are poorly understood. We studied the effect of warming and decreasing salinity on the diversity, functional traits, and biomass of benthic diatom and zooplankton communities in an indoor mesocosm experiment.

### 4.1 The Vulnerability of Organism Groups Belonging to Different Trophic Levels to the Effect of Warming and Decreasing Salinity (H1)

We hypothesized that zooplankton, that is, larger organisms and primary consumers, would be more vulnerable to climate change than benthic diatoms, that is, smaller organisms and primary producers. We based this hypothesis on the assumptions that larger and more active species, that is, zooplankton, may become unable to maintain their energetic requirements when facing high temperatures (da Silva et al. 2023), and that smaller organisms, that is, benthic diatoms, have higher diversity, which contributes to high functional resilience and protection against climate change effects (Duffy et al. 2016). However, our results did not support our hypothesis. Warming affected the species and trait composition of communities of both organism groups, whereas the interaction of simultaneous stressors, that is, warming and decreasing salinity, affected benthic diatoms but not zooplankton.

We think that there are a few possible reasons for the difference between our hypothesis and results. First, our four‐week‐long experiment was long enough for revealing the effects of environmental change on benthic diatom communities because their generation time is short, but the experiment may have been too short for revealing effects on zooplankton communities that include genera with considerably longer generation times (Hahn and Brennan 2024). Second, our warming scenario of five degrees centigrade was stressful for diatom communities, but seemingly beneficial for zooplankton, for example, as nauplii were more abundant in +17.6°C, as egg hatching rate may have been higher (cf., Vehmaa et al. 2012). And third, brackish‐water zooplankton communities in the northern Baltic Sea are fairly well adapted to salinity levels that are naturally present in this area, that is, salinity levels ca. 3–6 (von Weissenberg et al. 2022), whereas salinity ca. 5 is a threshold for benthic diatom communities (Remane 1934; Virta and Hedberg 2024). Thus, differences among salinity treatments in our experiment were appropriate for showing the effect of salinity decrease on benthic diatom communities but may have been too small for showing the effects of salinity decrease on zooplankton communities. Warming in our experiment was created by a temperature increase of five degrees centigrade, which has been found to be a typical peak anomaly of temperature during sea surface heat waves globally and in the Baltic Sea during recent years (Laufkötter et al. 2020; Rutgersson et al. 2022). This kind of a sudden exposure to higher temperature is likely to affect the structure and functioning of marine communities in benthic and pelagic realms by inducing, for example, species dominance shifts, size shifts, and metabolic changes (Brodeur et al. 2019; Göbeler et al. 2025). In our work, warming altered the species and trait composition of communities and dominating species both in the benthic diatom and zooplankton communities. In the benthic diatom communities, warming led to an increase of species that tolerate a wide variety of environmental conditions, such as *Achnanthidium minutissimum* (Siver et al. 2005), whereas the zooplankton communities shifted from rotifer‐dominated to copepod‐dominated. Traitwise, warming led to the proportional increase of diatoms that were small‐sized, non‐colony‐forming, non‐attaching, motile, and low‐profile, whereas the proportional abundances of large‐sized, colony‐forming, and high‐profile diatoms decreased. The decrease of large diatom species in higher temperatures is in line with previous studies showing that increasing temperatures favor small species (Sheridan and Bickford 2011). A likely explanation to this pattern is that warming increases the metabolic demand of organisms, increasing their nutrient use efficiency and, thus, intensifying nutrient limitation of the system (Laidler 1984). The consequent low nutrient concentrations favor small species, because they have a better nutrient uptake efficiency due to their higher surface‐area‐to‐volume ratio (Kriest and Oschlies 2007). The decrease of high‐profile diatoms during stressful conditions, such as strong currents, high abundance of grazers, or high temperatures, has also been documented before (Passy 2007; Stenger‐Kovács et al. 2013; da Silva et al. 2019), and it has implications on benthic food webs, because high‐profile diatoms are the main food source for benthic grazers (Salo et al., unpublished data). In zooplankton communities, a similar trend of shifting body size was detected, as the body size changed towards smaller body sizes over time due to large numbers of nauplii hatched (c.f. Daufresne et al. 2009). There was also less zooplankton in the treatment with ambient temperature, which may be due to slower egg hatching rates (Katajisto 2006).

Based on Remane (1934), Whitfield et al. (2012) and Virta and Hedberg (2024), salinity 5 is a critical level for brackish‐water taxa, indicating that a further decline in salinity in the Baltic Sea may have a strong effect on biodiversity and species survival. In our study, the individual effect of salinity was not significant for benthic diatoms and zooplankton, but the temperature × salinity interaction significantly affected the composition of diatom communities. This could be a multiple stress effect, indicating that simultaneously occurring stress factors have a strong effect on organisms (Krishna et al. 2025). Especially for phytoplankton, warming combined with an additional factor is the most critical stress combination for microalgae that are highly sensitive to multiple stress (Krishna et al. 2025). However, we found no effect of salinity on zooplankton. Zooplankton communities are often controlled by salinity differences in the Baltic Sea (Hall and Lewandowska 2022 and references therein); therefore, we think that either the salinity differences in our experiment were too small, or the experiment was too short to reveal the effects of salinity on zooplankton.

The response time of benthic diatoms was faster than that of zooplankton. The effects of the changing environment could be seen in benthic diatom communities within 2 weeks (cf., Virta and Hedberg 2024), whereas zooplankton required 4 weeks. This was expected because of the rapid life cycle and reproduction rate of microscopic organisms, such as diatoms, compared to larger organisms. An individual species in the diatom community can double its population size within 24 h through cell division (Spaulding et al. 2021), whereas the generation time of the zooplankton species/genus that were present in our experiment varies between a few days and several weeks (Bosque et al. 2001; Hahn and Brennan 2024). Short generation time of diatoms leads to fast responses of community structure and function to environmental changes and also enables fast adaptation of individual species to changing conditions (Jin and Agustí 2018).

### 4.2 The Effect of Climate on the Diversity and Biomass of Communities (H2)

We hypothesized that warming and decreasing salinity would lead to a decrease in diversity and biomass of both benthic diatoms and zooplankton, and that the decrease would be stronger in the communities of higher trophic level organisms, that is, zooplankton. For benthic diatoms, our hypothesis was partly confirmed, as warming resulted in lower diversity, but salinity had no effect on diversity or biomass of diatoms. However, for zooplankton, our hypothesis was proven incorrect, as warming resulted in higher diversity, and salinity had no effect.

In the benthic diatom communities, diversity decreased in higher temperature, but the loss of species from communities was not severe. Rather, the main reason for lower diversity in higher temperature was a dominance shift (Engel et al. 2020). Communities in the ambient temperature treatment consisted of several abundant species and had high evenness, whereas communities in the elevated temperature were dominated by one specific species (*Halamphora coffeiformis*) and were low in evenness. The reduction in diatom biomass in elevated temperature has been documented before (Hicks et al. 2011; Cartaxana et al. 2015). Even though benthic diatoms are highly tolerant to short‐term temperature fluctuations in, for example, intertidal zones, long‐term increase in temperature is detrimental to their productivity and growth. The lack of effect of decreasing salinity on diatom diversity and biomass in our experiment was surprising, because the diversity of benthic diatoms has previously been found to increase when salinity decreases below the threshold level of 5 (Remane 1934; Whitfield et al. 2012; Virta et al. 2020).

Concerning zooplankton, Helenius et al. (2017) showed that zooplankton diversity was both temperature and salinity dependent, based on a field study in the archipelago. Their piece of work partly supports our findings that zooplankton biodiversity and biomass responded positively to elevated temperature. Mesocosm studies report that zooplankton richness neither changed in different salinities nor varied over time (Hall and Lewandowska 2022). In the current work, we neither found salinity effects in zooplankton. Mäkinen et al. (2017), among others, have concluded that Baltic Sea zooplankton, including small‐bodied copepods, are well adapted to brackish water, which reflects their wide tolerance to salinity.

## 5 Conclusions

We studied the simultaneous effect of warming and decreasing salinity on benthic diatom and zooplankton communities using a mesocosm approach and hypothesized that organisms belonging to a lower trophic level, that is, benthic diatoms, would be less vulnerable to changing environment than organisms belonging to a higher trophic level, that is, pelagic zooplankton. However, our results did not support this hypothesis as warming affected the species and functional trait composition of the communities of both taxa, whereas the interaction between warming and decreasing salinity affected benthic diatoms but not zooplankton. This study has narrowed the knowledge gap of the effect of multiple stressors on aquatic communities during a rapidly changing environment. Future work should focus on combined effects of different climate change‐related factors on functional trait structure of various trophic levels.

## Author Contributions

**Leena Virta:** conceptualization (lead), formal analysis (lead), funding acquisition (lead), investigation (equal), project administration (lead), validation (equal), visualization (lead), writing – original draft (equal). **Jonna Engström‐Öst:** investigation (equal), validation (equal), writing – original draft (equal).

## Funding

This work was supported by Koneen Säätiö, Emil Aaltosen Säätiö, and Biotieteiden ja Ympäristön Tutkimuksen Toimikunta (361936).

## Conflicts of Interest

The authors declare no conflicts of interest.

## Data Availability

The datasets generated for this study are archived in Zenodo open repository, https://doi.org/10.5281/zenodo.19567959.

## Appendix A

**TABLE A1:** Variation of temperature (°C) and salinity in the 10 mesocosms during the 4‐week‐long experiment.

Date	Treatment	Temperature	Salinity
**1.6.2023**	Temp 12.6, Sal 3	12.8	3.8
1.6.2023	Temp 12.6, Sal 4	12.8	4.13
1.6.2023	Temp 12.6, Sal 5	12.5	5.12
1.6.2023	Temp 12.6, Sal 6	12.8	6.18
1.6.2023	Temp 12.6, Sal 7	12.8	7.16
1.6.2023	Temp 17.6, Sal 3	16.8	3.8
1.6.2023	Temp 17.6, Sal 4	16.7	4.2
1.6.2023	Temp 17.6, Sal 5	16.6	5.27
1.6.2023	Temp 17.6, Sal 6	16.9	6.26
1.6.2023	Temp 17.6, Sal 7	16.5	7.45
**7.6.2023**	Temp 12.6, Sal 3	12.8	3.85
7.6.2023	Temp 12.6, Sal 4	13	4.24
7.6.2023	Temp 12.6, Sal 5	12.5	5.26
7.6.2023	Temp 12.6, Sal 6	12.8	6.14
7.6.2023	Temp 12.6, Sal 7	12.7	7.19
7.6.2023	Temp 17.6, Sal 3	17	3.84
7.6.2023	Temp 17.6, Sal 4	16.6	4.31
7.6.2023	Temp 17.6, Sal 5	16.9	5.26
7.6.2023	Temp 17.6, Sal 6	17.3	6.3
7.6.2023	Temp 17.6, Sal 7	16.5	7.45
**14.6.2023**	Temp 12.6, Sal 3	13.2	3.85
14.6.2023	Temp 12.6, Sal 4	13.7	4.25
14.6.2023	Temp 12.6, Sal 5	12.8	5.28
14.6.2023	Temp 12.6, Sal 6	13	6.14
14.6.2023	Temp 12.6, Sal 7	13	7.23
14.6.2023	Temp 17.6, Sal 3	17.4	3.92
14.6.2023	Temp 17.6, Sal 4	16.9	4.49
14.6.2023	Temp 17.6, Sal 5	17.2	5.39
14.6.2023	Temp 17.6, Sal 6	17.7	6.44
14.6.2023	Temp 17.6, Sal 7	16.8	7.61
**21.6.2023**	Temp 12.6, Sal 3	13.3	3.83
21.6.2023	Temp 12.6, Sal 4	13.9	4.13
21.6.2023	Temp 12.6, Sal 5	12.9	5.15
21.6.2023	Temp 12.6, Sal 6	13.1	6.16
21.6.2023	Temp 12.6, Sal 7	13.2	7.14
21.6.2023	Temp 17.6, Sal 3	17.8	3.86
21.6.2023	Temp 17.6, Sal 4	17.1	4.48
21.6.2023	Temp 17.6, Sal 5	17.5	5.39
21.6.2023	Temp 17.6, Sal 6	18.1	6.33
21.6.2023	Temp 17.6, Sal 7	17	7.6

**TABLE A2:** Nutrient concentrations in the mesocosms in the beginning and end of the experiment.

Treatment	Start (May 26, 2023)		End (June 21, 2023)	
	${\mathrm{NO}}_{3}^{-}$ + ${\mathrm{NO}}_{2}^{-}$ (μg/L)	${\mathrm{PO}}_{4}^{3-}$ (μg/L)	${\mathrm{NO}}_{3}^{-}$ + ${\mathrm{NO}}_{2}^{-}$ (μg/L)	${\mathrm{PO}}_{4}^{3-}$ (μg/L)
Temp 12.6, Sal 3	30.5	< 3 (1.6)	< 3	< 3
Temp 12.6, Sal 4	31.8	< 3 (1.6)	< 3	< 3
Temp 12.6, Sal 5	35	< 3 (1.4)	< 3	< 3
Temp 12.6, Sal 6	33	< 3 (1.4)	< 3	< 3
Temp 12.6, Sal 7	35.9	< 3 (1.3)	< 3	< 3
Temp 17.6, Sal 3	28.3	< 3 (1.1)	< 3	< 3
Temp 17.6, Sal 4	33.5	< 3	< 3	< 3
Temp 17.6, Sal 5	32.1	< 3 (1.6)	< 3	< 3
Temp 17.6, Sal 6	32.3	< 3	< 3	< 3
Temp 17.6, Sal 7	38.9	< 3 (1.6)	< 3	< 3

*Note:* Values with < denote values that were under the official detection limit of the analyzer.
